# New Treatment for Alzheimer's Disease, Kamikihito, Reverses Amyloid-**β**-Induced Progression of Tau Phosphorylation and Axonal Atrophy

**DOI:** 10.1155/2014/706487

**Published:** 2014-02-23

**Authors:** Hidetoshi Watari, Yutaka Shimada, Chihiro Tohda

**Affiliations:** ^1^Division of Neuromedical Science, Department of Bioscience, Institute of Natural Medicine, University of Toyama, 2630 Sugitani, Toyama 930-0194, Japan; ^2^Department of Japanese Oriental Medicine, Graduate School of Medicine and Pharmaceutical Sciences, University of Toyama, 2630 Sugitani, Toyama 930-0194, Japan

## Abstract

*Aims.* We previously reported that kamikihito (KKT), a traditional Japanese medicine, improved memory impairment and reversed the degeneration of axons in the 5XFAD mouse model of Alzheimer's disease (AD). However, the mechanism underlying the effects of KKT remained unknown. The aim of the present study was to investigate the mechanism by which KKT reverses the progression of axonal degeneration. *Methods.* Primary cultured cortical neurons were treated with amyloid beta (A**β**) fragment comprising amino acid residues (25–35) (10 **μ**M) in an *in vitro* AD model. KKT (10 **μ**g/mL) was administered to the cells before or after A**β** treatment. The effects of KKT on A**β**-induced tau phosphorylation, axonal atrophy, and protein phosphatase 2A (PP2A) activity were investigated. We also performed an *in vivo* assay in which KKT (500 mg/kg/day) was administered to 5XFAD mice once a day for 15 days. Cerebral cortex homogenates were used to measure PP2A activity. *Results.* KKT improved A**β**-induced tau phosphorylation and axonal atrophy after they had already progressed. In addition, KKT increased PP2A activity *in vitro* and *in vivo*. *Conclusions.* KKT reversed the progression of A**β**-induced axonal degeneration. KKT reversed axonal degeneration at least in part through its role as an exogenous PP2A stimulator.

## 1. Introduction

Nearly 36 million people worldwide are living with dementia, and as the population ages, this number is expected to be more than triple by 2050. The countries with the largest number of people with dementia are China (5.4 million), Japan (4.3 million), the United States (3.9 million), India (3.7 million), Germany (1.5 million), Russia (1.2 million), France (1.1 million), Italy (1.1 million), and Brazil (1.0 million). Dementia has become a prominent social issue [1, 2]. Alzheimer's disease (AD), the most common cause of dementia, is characterized by extracellular deposits of amyloid beta (A*β*) protein and intracellular neurofibrillary tangles (NFT) composed of hyperphosphorylated tau. Acetylcholinesterase inhibitors (donepezil, galantamine, and rivastigmine) and an N-methyl-d-aspartate (NMDA) receptor antagonist (memantine) are currently the only approved drugs for the treatment of AD [3]. However, because these drugs do not target A*β* and hyperphosphorylated tau, it is difficult to prevent, halt, or reverse the disease [4].

In spite of efforts to discover drugs targeting A*β* and hyperphosphorylated tau, no truly effective therapy has been developed to date [5]. Solanezumab, a humanized monoclonal antibody to A*β* (16–24) that preferentially binds soluble A*β*, is one of the most notable immunotherapeutic agents for AD. In the phase II clinical trials, solanezumab increased plasma and CSF levels of A*β*40 and A*β*42, thus suggesting that the amount of A*β* plaques was decreased in the brain; however, solanezumab had no effect on behavioral tests of AD patients [6]. These results suggest that the reduction of A*β* levels after the appearance of symptoms is not effective for the treatment of AD. Other therapeutic approaches, such as tau aggregation inhibitors and anti-inflammatory drugs, are currently under investigation, but such efforts are on a very small scale. We propose that it is important for new anti-AD drugs to reverse the degeneration of neuronal networks after the appearance of symptoms.

From this point of view, we are investigating drugs used in Japanese traditional medicine (called Kampo), focusing on kamikihito (KKT). KKT is a herbal drug known for its effectiveness in treating insomnia, loss of appetite, amnesia, and depression. We previously reported the effect of KKT in the 5XFAD mouse model of AD [7]. 5XFAD mice exhibit increased A*β* production, A*β* plaques, and gliosis at 2 months of age and synapse loss and memory impairment at 4 months of age [8]. Therefore, 5XFAD mice are considered to be a model of AD after the appearance of symptoms. Our previous study showed that the administration of KKT to 5XFAD mice (4–7 months old) for 15 days improves memory impairment and reverses the degeneration of cortical axons and presynaptic terminals. These effects could be beneficial for AD patients, but the mechanism underlying the effects of KKT remains unknown. The aim of the present study is to investigate the mechanism by which KKT reverses progressive axon degeneration.

## 2. Materials and Methods

All experiments were performed in accordance with the Guidelines for the Care and Use of Laboratory Animals of the University of Toyama.

### 2.1. Preparation of KKT Extract

KKT is composed of 14 crude drugs: Ginseng Radix (*P. ginseng* C.A. Meyer), Polygalae Radix (*P. tenuifolia* Willd.), Astragali Radix (*A. membranaceus *Bunge), Zizyphi Fructus (*Zizyphus jujube* Mill. var. *inermis* Rehd.), Zizyphi Spinosi Semen (*Z. jujube* Mill. var. *spinosa*), Angelicae Radix (*Angelica acutiloba* Kitagawa), Glycyrrhizae Radix (*Glycyrrhiza uralensis* Fisch), Atractylodis Rhizoma (*Atractylodes japonica* Koidzumi ex Kitamura), Zingiberis Rhizoma (*Zingiber officinale* Roscoe), Poria (*Poria cocos* Wolf), Saussureae Radix (*Saussurea lappa* Clarke), Longanae Arillus (*Euphoria longana* Lam.), Bupleuri Radix (*Bupleurum falcatum* Linne), and Gardeniae Fructus (*Gardenia jasminoides* Ellis) at a ratio of 3 : 1.5 : 2: 1.5 : 3: 2 : 1 : 3 : 1 : 3 : 1 : 3 : 3 : 3, respectively. All crude drugs were purchased from Tochimoto Tenkaido (Osaka, Japan). The mixture of crude drugs (100 g) was extracted with water (500 mL) at 100°C for 50 min, evaporated under reduced pressure, and freeze-dried to obtain a powder extract (12.3 g).

### 2.2. Primary Culture

Embryos were removed from a pregnant ddY mouse (Japan SLC, Shizuoka, Japan) at 14 days of gestation. The cerebral cortices were dissected, and the dura mater was removed. The tissues were minced, dissociated, and cultured with Neurobasal medium (Gibco BRL, Rockville, MD, USA) containing 12% horse serum, 0.6% D-glucose, and 2 mM L-glutamine. Cells were grown in 8-well chamber slides (Falcon, Franklin Lakes, NJ, USA) or 10 cm dishes coated with 5 *μ*g/mL poly-D-lysine at 37°C in a humidified incubator with 10% CO_2_. On the next day of culture, the medium was replaced with fresh Neurobasal medium containing 2% B-27 supplement rather than horse serum.

### 2.3. A*β*-Induced Axonal Atrophy

Mouse cortical neurons (E14) were cultured in 8-well chamber slides (Falcon, Franklin Lakes, NJ, USA) at a density of 1.45 × 10^4^ cells/cm^2^ for 3 days. To induce axon atrophy, the cells were treated with A*β* (25–35) (10 *μ*M) (Sigma-Aldrich, St. Louis, MO, USA). The A*β* (25–35), an active fragment of A*β*, was previously incubated for 4 days at 37°C to induce aggregation. In experiments involving pretreatment, KKT (10 *μ*g/mL), Kenpaullon (KPL, 1 *μ*M, an inhibitor of glycogen synthase kinase-3*β*: GSK-3*β*), or vehicle was administered to the cells, and A*β* (25–35) was added to the cells 1 h later. At 24 h after incubation with A*β*, the medium was replaced with A*β* (25–35)-free medium, and the drug treatments were continued for an additional 72 h. In experiments involving posttreatment, cells were incubated with A*β* (25–35) (10 *μ*M) for 24 h. The medium was then replaced to remove A*β* (25–35). The replacement medium contained KKT (10 *μ*g/mL), KPL (1 *μ*M), or vehicle. After drug treatment for 72 h, the cells were fixed with 4% paraformaldehyde and immunostained axons with a monoclonal antibody against phosphorylated NF-H (pNF-H, dilution 1 : 500, Sternberger Monoclonals, Lutherville, MD, USA) and neuronal cell bodies with a polyclonal antibody against MAP 2a and 2b (MAP2, dilution 1 : 500, Chemicon, Temecula, CA, USA). Secondary antibodies were Alexa Fluor 488-conjugated goat anti-mouse IgG (dilution 1 : 300) and Alexa Fluor 568-conjugated goat anti-rabbit IgG (dilution 1 : 300) (Molecular Probes, Eugene, OR, USA). Fluorescent images were detected by a fluorescence microscope system (BX-61/DP70, Olympus, Tokyo, Japan). The lengths of axons were measured using the Neurocyte image analyzer (Kurabo, Osaka, Japan), which automatically traced and measured neurite lengths without measuring cell bodies. The total length of the axons was divided by the number of cells in an identical area to calculate the average length of axon per neuron.

### 2.4. A*β*-Induced Tau Phosphorylation

Mouse cortical neurons (E14) were cultured in 8-well chamber slides at a density of 1.45 × 10^4^ cells/cm^2^ for 3 days. For pretreatment experiments, KKT (10 *μ*g/mL), KPL (1 *μ*M), or vehicle was administered to the cells, and A*β* (25–35) (10 *μ*M) was added 1 h later. At 4 h or 72 h after incubation with A*β*, the cells were fixed. For posttreatment experiments, cells were treated with A*β* (25–35) (10 *μ*M) for 24 h. Then, the medium was replaced with medium lacking A*β* (25–35). KKT (10 *μ*g/mL), KPL (1 *μ*M), or vehicle was included in the replacement medium. At 72 h after drug treatment, the cells were fixed and immunostained with a monoclonal antibody against paired helical filament tau (PHF-tau, AT-8 clone, dilution 1 : 200, Thermo Scientific, Rockford, IL, USA). Alexa Fluor 488-conjugated goat anti-mouse IgG (dilution 1 : 300) was used as the secondary antibody. Images were captured using a fluorescence microscope system (BX-61/DP70, Olympus). The level of tau phosphorylation was measured as the fluorescence intensity in the cell body of each neuron using a CS analyzer (ATTO, Tokyo, Japan).

### 2.5. PP2A Activity Assay

Mouse cortical neurons (E14) were cultured in a 10 cm dish at a density of 8.67 × 10^4^ cells/cm^2^ for 3 days. KKT (10 *μ*g/mL) or vehicle was administered to the cells, and A*β* (25–35, 10 *μ*M) was added 1 h later. At 72 hours after incubation with A*β*, the cells were harvested and suspended in phosphate storage buffer (2 mM EGTA, 5 mM EDTA, 0.5 mM PMSF, 150 mM NaCl, 50 mM Tris-HCl (pH 7.4), 1% Triton X-100, and protease inhibitor cocktail (1 : 200)). The cell lysates were sonicated and centrifuged at 16,000 ×g for 10 min, and the supernatants were used for the PP2A activity assay. PP2A activity was measured using a serine/threonine phosphatase assay system (V2460, Promega, Madison, WI, USA) according to the manufacturer's protocol. The cell lysates were passed twice through Sephadex G-25 Spin Columns to remove endogenous free phosphate. The protein concentration was determined using the Pierce 660 nm Protein Assay. PP2A reaction buffer (50 mM imidazole, pH 7.2, 0.2 mM EGTA, 0.02% *β*-mercaptoethanol, and 0.1 mg/mL bovine serum albumin) and 1 mM phosphopeptide were prepared in a 96-well plate. The enzyme reaction was started by adding 2.5 *μ*g of the protein lysate to each well, and the mixture was incubated for 30 min at 33°C. The reaction was stopped by the addition of 50 *μ*L of molybdate dye-additive mixture, and the plate was incubated at room temperature for 15 min. Phosphate release from the substrate was detected by measuring the absorbance of the molybdate-malachite green-phosphate complex at 630 nm.

### 2.6. Animals

Transgenic mice (5XFAD) [8] were obtained from Jackson laboratory (Bar Harbor, ME, USA). To investigate the effect of KKT on 5XFAD mice, the study used hemizygous 5XFAD mice (female, 9–11 months) and nontransgenic wild-type mice (female, 9–10 months). All mice were housed with free access to food and water and kept in a controlled environment (22 ± 2°C, 50 ± 5% humidity, 12 h light cycle starting at 7:00 a.m.).

KKT was dissolved in physiological saline. KKT (500 mg/kg/day) or vehicle solution was intragastrically administered once a day for 15 days. On the sixteenth day, the mice were sacrificed, and the cerebral cortex was isolated and homogenized in phosphate storage buffer for the PP2A activity assay.

### 2.7. GSK-3*β* Activity Assay

Mouse cortical neurons (E14) were cultured in 8-well chamber slides at a density of 1.45 × 10^4^ cells/cm^2^ for 3 days. KKT (10 *μ*g/mL) or vehicle was administered to the cells, and A*β* (25–35) (10 *μ*M) was added 1 h later. At 4 h after incubation with A*β*, the cells were fixed with 4% paraformaldehyde and immunostained with a monoclonal antibody against phosphorylated GSK-3*β* at Tyr-216 (pY216, dilution 1 : 200, BD Biosciences, Franklin Lakes, NJ, USA) as a marker of activated GSK-3*β* and a polyclonal antibody against GSK-3*β* (dilution 1 : 500, Santa Cruz Biotechnology, Santa Cruz, CA, USA) as a marker of total GSK-3*β*. Alexa Fluor 488-conjugated goat anti-mouse IgG (dilution 1 : 300) and Alexa Fluor 568-conjugated goat anti-rabbit IgG (dilution 1 : 300) were used as secondary antibodies. Images were captured using a fluorescence microscope system (BX-61/DP70, Olympus). Expression of phosphorylated GSK-3*β* and total GSK-3*β* was measured by determining the fluorescence intensity in the cell body of each neuron using a CS analyzer (ATTO). The ratio of GSK-3*β* (pY216) to GSK-3*β* (total) was calculated.

### 2.8. Statistical Analysis

Statistical comparisons were performed through a One-way analysis of variance (ANOVA) with post hoc Dunnett's test or Bonferroni's multiple comparison test using GraphPad Prism 5 (GraphPad Software, La Jolla, CA, USA). *P* < 0.05 was considered significant. The data are presented as the means ± SE.

## 3. Results

### 3.1. KKT Decreases A*β* (25–35)-Induced Phosphorylation of Tau

We first focused on the effectiveness of KKT against A*β* (25–35)-induced phosphorylation of tau. Full-length A*β* (1–42) and its active fragment A*β* (25–35) have been reported to induce phosphorylation of tau [9, 10]. Phosphorylation of tau leads to axon transport deficits, synaptic loss, and mitochondrial dysfunction, resulting in axonal dysfunction [11–13]. Among the various kinases involved in phosphorylation of tau, GSK-3*β* plays a critical role in AD pathology [14]. We therefore investigated the effect of KKT (10 *μ*g/mL) and KPL (inhibitor of GSK-3*β*; Ki = 0.23 *μ*M) (1 *μ*M) on A*β* (25–35)-induced phosphorylation of tau. Drugs were administered before or after treatment with A*β* (25–35).

**Figure 1:**
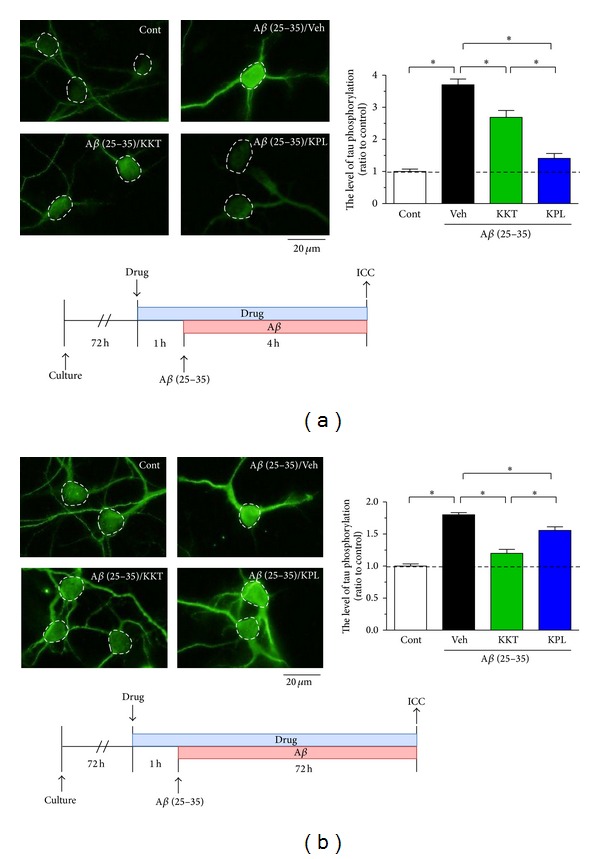
Effect of KKT and KPL pretreatment on A*β*-induced phosphorylation of tau. Cortical neurons were cultured for 3 days and then treated with vehicle solution, KKT (10 *μ*g/mL), or KPL (1 *μ*M). At 1 h after each treatment, the cells were treated with A*β* (25–35) (10 *μ*M) for 4 h (a) or 72 h (b). After A*β* treatment, the cells were fixed and immunostained with an antibody against paired helical filament tau (PHF-tau). The PHF-tau fluorescence intensity was quantified for each treatment. *P < 0.05 (*n* = 15–20, One-way ANOVA, post hoc Bonferroni's multiple comparison test).

As shown in Figure 1(a), treatment of cortical neurons with A*β* (25–35) for 4 h resulted in a 3.7-fold increase in phosphorylation of tau compared with the control. However, the phosphorylation of tau was attenuated to 2.6-fold by 1 h of pretreatment with KKT (10 *μ*g/mL). With KPL pretreatment, phosphorylation of tau was remarkably prevented with 1.4-fold (Figure 1(a)). Tau remained phosphorylated at 72 h after A*β* (25–35) treatment, although the degree of phosphorylation was lower than at 4 h after treatment (Figure 1(b)). Phosphorylation of tau in the late phase was remarkably prevented by pretreatment with KKT, but pretreatment with KPL resulted in only a slight attenuation of tau phosphorylation.

**Figure 2:**
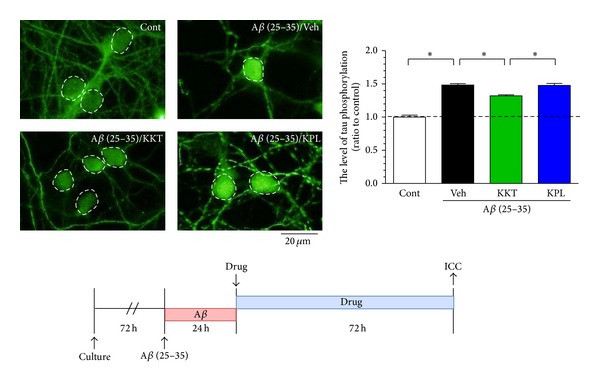
Effect of KKT and KPL posttreatment on A*β*-induced phosphorylation of tau. Cortical neurons were cultured for 3 days and then treated with A*β* (25–35) (10 *μ*M). At 24 h after A*β* treatment, A*β* was removed from the medium, and the cells were treated with vehicle solution, KKT (10 *μ*g/mL), or KPL (1 *μ*M). At 72 h after each treatment, the cells were fixed and immunostained with an antibody against paired helical filament tau (PHF-tau). The PHF-tau fluorescence intensity was quantified for each treatment. *P < 0.05 (*n* = 15–17, One-way ANOVA, post hoc Bonferroni's multiple comparison test).

We next investigated the effects of posttreatment with KKT (10 *μ*g/mL) and KPL (1 *μ*M) on A*β* (25–35)-induced phosphorylation of tau (Figure 2). We added A*β* (25–35) to the cells for 24 h, and the cells were incubated with each drug for the next 72 h without A*β* (25–35). KPL no longer affected the A*β*-induced phosphorylation of tau under these conditions, whereas KKT posttreatment significantly inhibited the phosphorylation of tau.

These results suggest that KKT reduces the phosphorylation of tau at both early and late phases and that increased tau phosphorylation can be reversed by KKT. In contrast, KPL may inhibit the phosphorylation of tau only in the early phase.

### 3.2. KKT Reverses A*β* (25–35)-Induced Axonal Atrophy

Figure 1 shows that KKT remarkably reduced the phosphorylation of tau in the late phases (Figure 1(b)), and KPL prevented the phosphorylation of tau more completely in the early phases (Figure 1(a)). Inhibition of tau phosphorylation by KPL no longer occurred when phosphorylation had already progressed (Figure 2). Because it is known that A*β* induces axonal atrophy through phosphorylation of tau, we investigated the effects of KKT and KPL on A*β* (25–35)-induced axonal atrophy.

**Figure 3:**
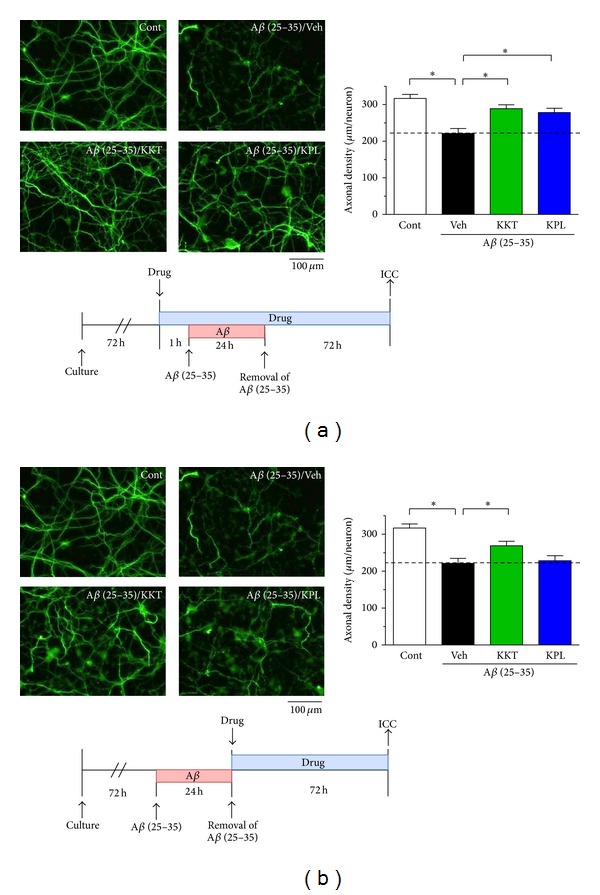
Effect of KKT and KPL pre- and posttreatment on A*β*-induced axonal atrophy. (a) For pretreatment, cortical neurons were cultured for 3 days and then treated with vehicle solution, KKT (10 *μ*g/mL), or KPL (1 *μ*M). After 1 h, the cells were treated with A*β* (25–35) (10 *μ*M) for 24 h, and A*β* was removed from the medium. Each drug treatment was continued for 72 h. (b) For posttreatment, cortical neurons were cultured for 3 d and then treated with A*β* (25–35) (10 *μ*M). At 24 h after A*β* treatment, A*β* was removed from the medium and the cells were treated with vehicle solution, KKT (10 *μ*g/mL), or KPL (1 *μ*M) for 72 h. After each drug treatment, the cells were fixed and immunostained with an antibody against phosphorylated NF-H and MAP2. The lengths of phosphorylated NF-H-positive neurites were quantified for each treatment. *P < 0.05 (*n* = 23–31, One-way ANOVA, post hoc Bonferroni's multiple comparison test).

Treatment of cortical neurons with A*β* (25–35) (10 *μ*M) for 24 h significantly decreased the axonal density (Figure 3(a)), which did not recover even 72 h after the removal of A*β* (25–35) (Figure 3(b)). In the pretreatment protocol, administration of KKT (10 *μ*g/mL) and KPL (1 *μ*M) significantly inhibited A*β* (25–35)-induced axonal atrophy (Figure 3(a)). The protection of axonal atrophy was equivalent in neurons that were pretreated with KKT or with KPL. In contrast, in the posttreatment protocol, only administration of KKT reversed A*β* (25–35)-induced axonal atrophy (Figure 3(b)). These results suggest that KKT, but not KPL, increases axonal density even after axonal atrophy has already progressed.

### 3.3. KKT Reverses the A*β*-Induced Decrease in PP2A Activity

**Figure 4:**
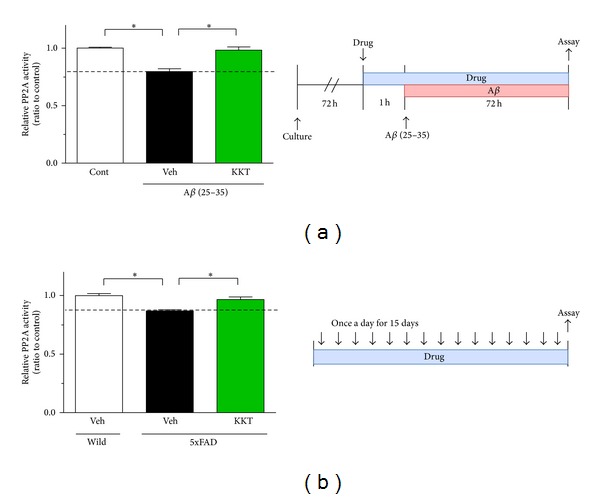
KKT enhances PP2A activity *in vitro* and *in vivo*. (a) Cortical neurons were cultured for 3 d and then treated with vehicle solution or KKT (10 *μ*g/mL). At 1 h after each treatment, the cells were treated with A*β* (25–35) (10 *μ*M) for 72 h. The cell lysates were prepared for the PP2A activity assay. PP2A activity was measured using a serine/threonine phosphatase assay system. (b) Vehicle solution or KKT (500 mg/kg/day) was administered once a day for 15 days to wild-type or 5XFAD mice. Cerebral cortex homogenates were used for the PP2A activity assay. *P < 0.05 versus Veh (*n* = 4–8, One-way ANOVA, post hoc Dunnett's test).

KKT remarkably reduced the phosphorylation of tau in the late phase (Figure 1(b)). To explain the long-lasting inhibitory effect of KKT on the phosphorylation of tau, we proposed that KKT may play a role in the dephosphorylation of tau. It was previously reported that A*β* increases the phosphorylation of tau by decreasing PP2A activity [15]; we therefore investigated the effect of KKT on PP2A activity under A*β* (25–35) treatment. KKT (10 *μ*g/mL) was added to the cells, and A*β* (25–35) (10 *μ*M) was added 1 h later. PP2A activity was measured 72 h after A*β* addition. As shown in Figure 4(a), A*β* treatment decreased PP2A activity to 79.6% compared with the control group. However, pretreatment with KKT completely prevented the decrease in PP2A activity (Figure 4(a)).

PP2A activity was also measured* in vivo*. KKT (500 mg/kg/day) was administered orally to the 5XFAD mice, and cerebral cortex lysates were prepared for measurement of PP2A activity. Our previous data showed that KKT administration for 15 days completely restored memory impairment in 5XFAD mice [7]. In the present experiment, we further confirmed that memory was improved by KKT treatment (data not shown). Vehicle-treated 5XFAD mice showed a significant decrease in PP2A activity compared with wild-type mice. However, KKT treatment of 5XFAD mice significantly increased PP2A activity compared with vehicle treatment (Figure 4(b)). These results demonstrate that KKT enhances the activity of PP2A under A*β* (25–35)-induced downregulation of PP2A activity.

### 3.4. KKT Has No Effect on GSK-3*β* Activity

**Figure 5:**
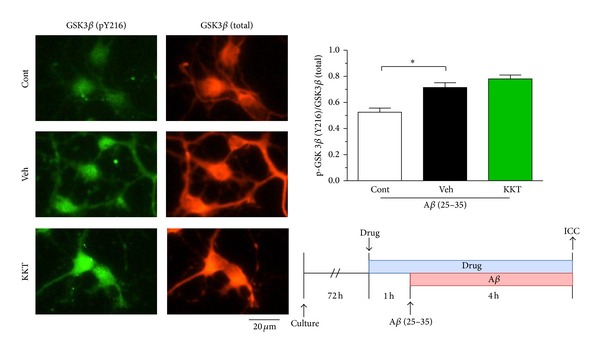
Effect of KKT on A*β*-induced activation of GSK-3*β*. Cortical neurons were cultured for 3 d and then treated with vehicle solution or KKT (10 *μ*g/mL). At 1 h after each treatment, the cells were treated with A*β* (25–35) (10 *μ*M) for 4 h. After A*β* treatment, the cells were fixed and immunostained with antibodies against phosphorylated GSK-3*β* at Tyr-216 (pY216), a marker of activated GSK-3*β*, and against total GSK-3*β*. The fluorescence intensity was quantified for each treatment. The ratio of GSK-3*β* (pY216) to GSK-3*β* (total) is shown. *P < 0.05 versus Veh (*n* = 15-16, One-way ANOVA, post hoc Dunnett's test).

Figures 1 and 2 show that KKT inhibits the A*β* (25–35)-induced phosphorylation of tau in both the early and late phases. However, the effect of KKT in the early phase was lower than that of KPL. Because the effects of KKT and KPL were different, we next investigated whether KKT was also an inhibitor of GSK-3*β* or not. GSK-3*β* activity was shown to be regulated by phosphorylation at specific residues; for example, phosphorylation of Ser9 and Ser389 inhibited its kinase activity, whereas phosphorylation at Tyr216 was required for its activation [16]. In this experiment, we measured the ratio of GSK-3*β* (pY216) to GSK-3*β* (total) to evaluate the level of GSK-3*β* activation. As shown in Figure 5, A*β* (25–35) (10 *μ*M) treatment for 4 h significantly increased the amount of phosphorylated GSK-3*β* (Tyr216). However, pretreatment with KKT (10 *μ*g/mL) had no effect on this increase (Figure 5). These results suggest that KKT may not be involved in the regulation of GSK-3*β* activity.

## 4. Discussion

Senile plaques and neurofibrillary tangles are two hallmark pathologies of AD. Senile plaques contain extracellular deposits of fibrillar A*β*, and neurofibrillary tangles are composed of hyperphosphorylated tau. It has been reported that A*β* induces phosphorylation of tau [9, 10] and that the phosphorylation of tau leads to axon transport deficits, synaptic loss, and mitochondrial dysfunction [11–13]. These disturbances eventually lead to axonal degeneration and neuronal death. Consequently, therapeutic approaches targeting tau have been growing steadily in recent years. For example, 2-methyl-5-(3-4-[(S)-methylsulfinyl]phenyl-1-benzofuran-5-yl)-1,3,4-oxadiazole (MMBO) and NP12 have been reported to decrease tau phosphorylation and reverse cognitive deficits in AD mouse models, which are novel GSK-3 inhibitors [17, 18]. In patients with mild to moderate AD, methylene blue (a tau aggregation inhibitor) is effective in treating cognitive deficits [19]. Phase II clinical trials of NP12 (tideglusib) and phase III clinical trials of LMTX, a derivative of methylene blue, are ongoing.

In this study, we investigate the mechanism underlying the effect of KKT. KKT reversed memory impairment in 5XFAD mice in our previous study [7], and here we demonstrate three properties of the effects of KKT. First, posttreatment with KKT reverses the progression of tau phosphorylation; however, posttreatment with KPL has no effect on tau phosphorylation (Figure 2). Second, posttreatment with KKT restores atrophied axons, whereas KPL does not (Figure 3(b)). Third, KKT enhances the activity of PP2A (Figure 4) but is not involved in the regulation of GSK-3*β* activity (Figure 5).

Recent reports of therapeutic strategies targeting A*β*, such as solanezumab and semagacestat, demonstrate that decreasing A*β* deposits or plasma A*β* levels after the appearance of symptoms does not improve cognitive dysfunction [6, 20]. In contrast, several reports have shown that tau is essential for A*β*-induced neuronal dysfunction *in vitro* and *in vivo* [21, 22]. Therefore, a decrease in tau phosphorylation may improve the symptoms of AD. From this point of view, it is very interesting that KKT reverses the progression of tau phosphorylation and axonal atrophy.

Our data suggest that KKT decreases the phosphorylation of tau through the activation of PP2A; however, the mechanism by which KKT increases PP2A activity remains unknown. The PP2A subtype accounts for more than 70% of all phosphatases in the human brain. In addition, the activity of PP2A decreases more than that of other phosphatases in the brains of AD patients [23]. Thus, PP2A likely plays a critical role in the dephosphorylation of tau. There are reports showing that PP2A activators decrease the phosphorylation of tau and reverse memory deficits in an AD model. For example, memantine enhances PP2A activity indirectly, and 1 year of treatment with memantine significantly decreases the levels of phosphorylated tau in the CSF of AD patients [24, 25]. Other reports have shown that sodium selenite, which induces dephosphorylation of tau via PP2A activation, improves memory deficits in tau transgenic mice [26]. Therefore, activation of PP2A likely plays an important role in the effects of KKT for AD treatment. However it has also been reported that KKT ameliorates scopolamine-induced spatial memory deficits and methyl-*β*-carboline-3-carboxylate(*β*-CCM)-induced-anxiogenic behavior [27, 28]. These reports suggest that KKT may stimulate the cholinergic system and antagonize the benzodiazepine receptor system. Multiple constituents of KKT may therefore contribute to its pharmacological effects.

Our present data indicating that KKT is effective even after A*β*-induced degeneration has occurred are important. Future studies should clarify which compounds in KKT are related to the restorative effect. KKT extract contains many constituents. Among them, at least ginsenoside Rg1, ginsenoside Rb1, saikosaponin a, saikosaponin b1, saikosaponin b2, geniposide, liquiritin, apioliquiritin, glycyrrhizic acid, 6-gingerol, and ligustilide were identified in our previous report [7]. Ginsenoside Rb1, a constituent in Ginseng Radix, attenuated spatial memory deficits in A*β* (25–35)-injected mice [29] and extended axons under A*β* (25–35) treatment (our unpublished data). Therefore ginsenoside Rb1 may be one of the active constituents in KKT.

Also we previously showed that posttreatment with Polygalae Radix extract reversed A*β* (25–35)-induced axonal atrophy [30]; therefore, Polygalae Radix may be at least partially responsible for the effect of KKT.

AD is characterized by two pathological hallmarks: A*β* aggregates and phosphorylated tau. However, AD is a multifactorial disease caused by protein misfolding and aggregation, oxidative stress, metal dyshomeostasis, altered protein phosphorylation, and mitochondrial dysfunction. One of the reasons for the failure of A*β*-targeted immune therapy in clinical trials is that the drugs have only one pharmacological target. In light of the multifactorial pathology of AD, it has been suggested that a multitarget drug, such as Memoquin, is necessary for AD treatment [31]. Memoquin acts as an acetylcholinesterase inhibitor, BACE1 inhibitor, and antioxidant, and it has been reported to improve scopolamine- and A*β*-induced cognitive impairment [32].

KKT has been used for a long time and is based on traditional philosophy (called Kampo in Japan) and offers the particular advantage of not being composed of only one or two active compounds. It is likely that the synergistic and additive effects of multiple compounds are necessary to obtain the best treatment performance. This multitarget treatment paradigm is inline with the concept of systems pharmacology [33].

## 5. Conclusion

KKT reverses axonal degeneration, in part through its action as an exogenous PP2A stimulator. In Japan, Korea, and China, KKT is already prescribed as a traditional formula for clinical medication. The usefulness of KKT as an anti-AD drug with the crucial property of reversing memory dysfunction must be confirmed by clinical studies.
